# Robust Wheel Detection for Vehicle Re-Identification

**DOI:** 10.3390/s23010393

**Published:** 2022-12-30

**Authors:** Sally Ghanem, Ryan A. Kerekes

**Affiliations:** Oak Ridge National Laboratory, Oak Ridge, TN 37830, USA

**Keywords:** object detection, vehicle re-identification, wheel detector

## Abstract

Vehicle re-identification is a demanding and challenging task in automated surveillance systems. The goal of vehicle re-identification is to associate images of the same vehicle to identify re-occurrences of the same vehicle. Robust re-identification of individual vehicles requires reliable and discriminative features extracted from specific parts of the vehicle. In this work, we construct an efficient and robust wheel detector that precisely locates and selects vehicular wheels from vehicle images. The associated hubcap geometry can hence be utilized to extract fundamental signatures from vehicle images and exploit them for vehicle re-identification. Wheels pattern information can yield additional information about vehicles in questions. To that end, we utilized a vehicle imagery dataset that has thousands of side-view vehicle collected under different illumination conditions and elevation angles. The collected dataset was used for training and testing the wheel detector. Experiments show that our approach could detect vehicular wheels accurately for 99.41% of the vehicles in the dataset.

## 1. Introduction

Extracting discriminating features is an essential part of target classification and re-identification (re-ID). The extracted features can be utilized to re-identify the target accurately and precisely. Accurate and efficient target representation is crucial for real time decision making. Recent developments in deep learning have provided many possibilities in developing efficient target re-ID models. Computer Vision has witnessed a wealth of great research activity in object detection, classification and re-ID, all with their strengths and limitations.

Vehicle re-ID has become a challenging problem in computer vision and intelligent transportation systems. Vehicle re-ID objective is to match a target vehicle across different captured vehicle images. Some of the challenges that face vehicle re-ID include lack of sufficient or realistic surveillance data, changes of viewpoints, illumination conditions, and occlusions. Due to the ongoing advancements in computing, neural network architectures, and dataset collection, real-time vehicle re-ID is now a realizable goal. Vehicle re-ID is a vital task in intelligent transportation systems due to its broad applicability. Vehicle re-ID methods can be utilized in many significant real-world applications including but not limited to suspicious vehicle search, road access restriction management, cross-camera vehicle tracking, traffic time estimation, toll collection, traffic behavior analysis, vehicle counting, access control, and border control.

Conventional vehicle re-identification methods such as license plate recognition and Radio frequency identification tags RFID [1,2] have been used for that purpose for a long time. Unfortunately, such license plate based methods can often be unreliable because of camera imperfections, occluded characters, and the absence of plates due to pose variations.

**Figure 1:**
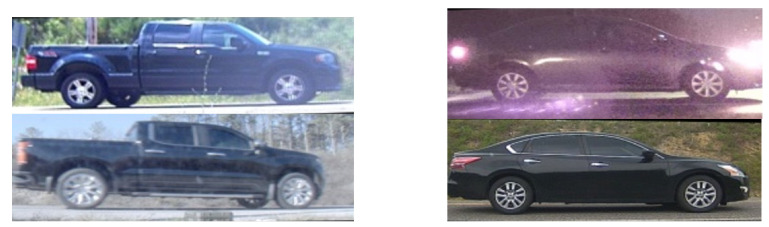
Similar looking vehicles with different wheels.

In this paper, we propose to develop a foundational capability that help precisely detect and extract wheels from vehicle images. Our approach will aid vehicle re-ID and tracking not only by enabling comparison of the wheels and their associated geometry, but also enabling precise alignment of an overall vehicle image by its wheel coordinates for subsequent comparison. To the best of our knowledge, this is the first work that discusses the topic of vehicular wheel detection/selection using a post processing approach. Developing a precise wheel detector will thus provide supplementary information and help to discriminate between very similar looking vehicles. As a result, similar looking vehicles could be distinguished if their hubcaps are distinct as shown in Figure 1. For that purpose, we utilized a large vehicle imagery dataset representing diverse conditions of illumination, backgrounds and elevation angles [3,4].

The rest of the paper is organized as follows, in Section 2, we describe related work. In Section 3, the dataset structure is demonstrated. In Section 4, we define our approach and present our experimental results, while Section 5 provides concluding remarks and summarizes future work.

## 2. Related Work

The topic of vehicle re-ID has been extensively studied in the literature using datasets collected by surveillance cameras. One of the key factors that challenges the performance of vehicle re-ID techniques is the lack of sufficient data. Some of the benchmark publicly available datasets include Veri-776 [5], BoxCars116k [6], VRIC [7], and VERI-Wild [8]. The Veri-776 [5] has been widely used by the computer vision community for vehicle re-ID applications. The majority of the publicly available datasets has non-overlapping viewpoints/poses with limited classes and instances for each class. Moreover, the same vehicle might look different due to unconstrained environment, camera calibration, location on the roadside, illumination changes, resolution variations, and different viewpoints. Data labeling can also be another challenge for vehicle re-ID methods since training a robust model in a supervised way is not achievable without a sufficient amount of labeled data. However, manually annotating a large dataset can be very expensive. Due to the aforementioned reasons and for the purpose of this paper, we will use the Profile Images and Annotations for Vehicle Re-identification Algorithms (PRIMAVERA) dataset [3]. The dataset contains more than 600,000 images for 13,000 vehicles where each vehicle has a different number of side-view images depending on the number of collected frames. The images were captured from different distances using various lenses and under different illumination conditions.

Recent developments in computer vision and deep learning have provided many possibilities in developing efficient Vehicle re-ID models. Fine grained extraction re-ID methods [9] can highlight the discriminative features in different spatial locations. Most vehicle re-ID models use Convolutional Neural Networks (CNNs) to automatically extract and optimize features [10,11,12]. In [10], a Region Aware deep Model (RAM) was proposed to embed the detailed visual cues and extract features from local regions. In addition, a learning algorithm was proposed to jointly exploit vehicle IDs, types/models, and colors to train their model. In [11], the authors proposed a two-stage network for vehicle re-identification utilizing visual and spatio-temporal information. A Spatial and Channel Attention Network (SCAN) based on Deep Convolutional Neural Network (DCNN) was proposed in [12] to automatically locate discriminative regions on vehicles. The proposed model is composed of two branches; spatial and channel attention branch to refine feature maps. In [13], part-regularized feature preserving method was introduced to enhance the ability of subtle discrepancies by utilizing different vehicle parts for detection; lights, windows, and vehicle brand. Moreover, RNN-based Hierarchical Attention classification model for vehicle re-identification was proposed in [14]. Their frameworks introduces coarse-to-fine category hierarchical dependency to detect visual appearance cues such as windshield stickers and customized paintings. In [4], a framework was proposed for decision fusion utilizing Siamese networks to extract discriminating features from vehicle images and their associated wheels.

Developing object detection capabilities has been a front and center topic in computer vision. Traditional object detection methods relies on handcrafted features, however, their performance can be further optimized. Due to the recent advances in deep learning, powerful models have been developed to extract deeper features, learn better data representations and optimize network architecture [15]. The performance of these deep object detection models can vary differently based on network architecture and optimization function. Object detection have widely been adopted in analyzing images for many computer vision applications. These application include human behavior analysis [16], image classification [17,18], autonomous driving [19,20], and face recognition [21].

Classification and regression-based object detection frameworks, such as YOLO [22] and Single Shot MultiBox Detector (SSD) [23], often map image pixels to bounding box coordinates and confidence scores. Instead of utilizing classifiers to perform detection, the YOLO model [22] applies regression to spatially separated bounding boxes and class probabilities. The YOLO neural network architecture can process images in real-time at 45 frames per second. A simpler version of YOLO can achieve 155 fps. Furthermore, YOLO can generate fewer false positives on the background. The improved model YOLOv2, proposed in [24], could detect over 9000 object categories and offered various enhancements to the YOLO detection method and utilized multi-scale training method. Although YOLO is one of the best models for object detection, it encounters difficulty in detecting smaller objects in groups due to strict spatial constraints imposed on bounding boxes. Moreover, YOLO struggles to generalize well with unfamiliar aspect ratios.

To overcome the aforementioned challenges and inspired by the success of Region Proposal Network (RPN) [25] and multiscale representation [26], SSD was proposed in [23]. The SSD model utilize a set of anchor boxes with various aspect ratios and scales. The model fuses predictions from multiple feature maps with different resolutions to detect varying size objects. SSD significantly outperforms other well-known object detection models in terms of accuracy. For input size 300 × 300, the model runs at 59 fps, which is more efficient than YOLO. In addition, SSD has much better accuracy compared to other single stage methods with smaller input image size.

The primary contribution of this paper is an automatic wheel detector that precisely selects wheel bounding boxes from vehicle images. A SSD network is retrained to provide the bounding boxes information for vehicles’ wheels. In addition, each detection is accompanied with a confidence score. In our evaluation, we will utilize the PRIMAVERA dataset [3]. Point well taken. To the best of our knowledge, this is the first work that provides a software for vehicle wheel detection and selection. To better explore the potential contribution of our proposed approach, we compare the performance of our framework with a conventional wheel detector. The conventional wheel detector (or the baseline method) selects the two wheels with highest confidence in each image in contrast to our method which selects the wheel pair using vehicle-specific post-processing algorithm that eliminates false detections.Experimental results confirm that our approach could precisely detect vehicular wheels under varying conditions of illumination and elevation angles.

## 3. Dataset Description

**Figure 2:**
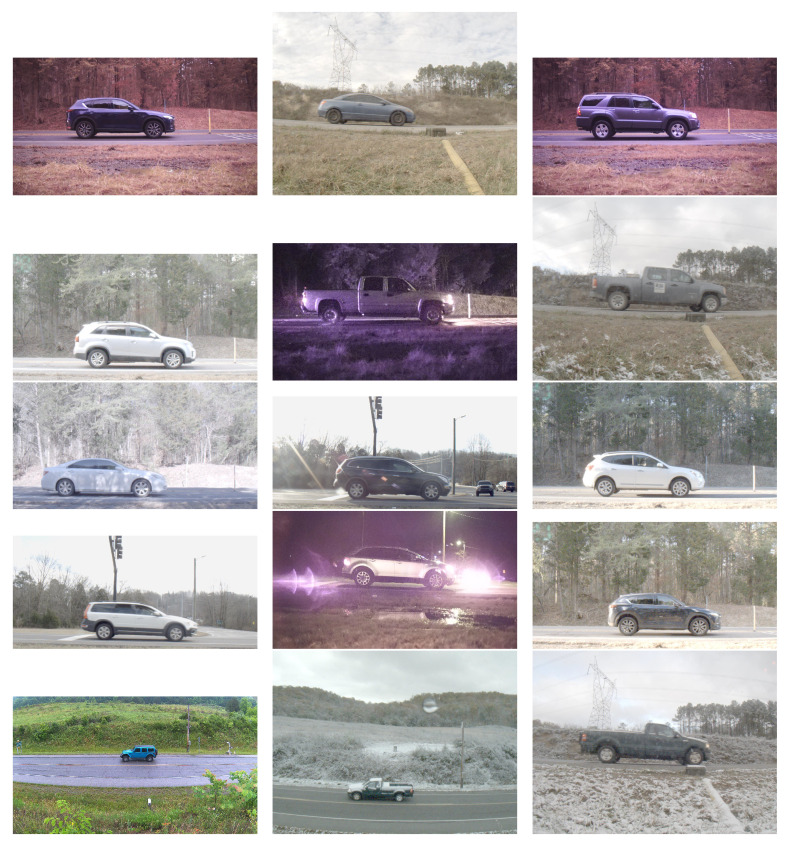
Sample images from the PRIMAVERA dataset.

To substantiate the validation of the proposed wheel detector model, we utilized a large dataset of vehicular imagery consisting of thousands of side-view images. The collected dataset was published in [3]. A roadside sensor system was employed to collect vehicle imagery using using various cameras and a radar unit. Side-view vehicle images were taken from distances ranging between 1 and 20 m using 1.8-mm to 6-mm lenses. Vehicle images were collected during both day and night over the course of several years. The road-side cameras were placed both at ground level and at elevated angles, providing a clear profile view of passing vehicles. The collections were done in speed zones ranging 25 to 45 MPH and near intersections where vehicles may have slowed or stopped. Moreover, license plate readers were employed to create a ground-truth label for each vehicle. Actual license plate numbers were replaced with a number and used as a label for the vehicle. In Figure 2, we show some sample images from the PRIMAVERA dataset.

The PRIMAVERA dataset has 636,246 images picturing 13,963 vehicles. Each vehicle has a different number of images depending on the number of times it passed by the cameras.

## 4. Experiments and Results

In this Section, we will elaborate on the structure of our wheel detector and evaluate its performance on the dataset.

### 4.1. Vehicle Detection

**Figure 3:**
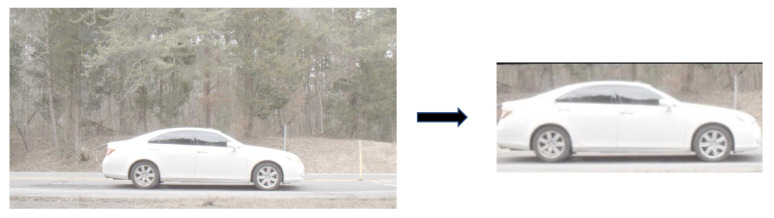
Before and after vehicle detection.

A vehicle detector was trained to detect a vehicle from video frames using a part of the dataset. The training data for the vehicle detector consisted of 543,926 images representing 11,918 vehicles. To that end, we retrained a SSD Mobilenet V2 network [27] to detect and locate vehicles using the training set. SSD is a single-shot model intended to perform object detection and implemented using the Caffe framework. The SSD model has been trained on the Common Objects in Context (COCO) image dataset [28]. The pre-trained SSD provides a good performance for locating the vehicle bounding box in each frame. The output of the vehicle detection network is a bounding box that provides the coordinates of the vehicle location in each image. We crop the vehicle image and resize it to 300×300 while preserving the aspect ratio. Specifically, we re-scale the bounding box coordinates such that it corresponds to a square around the vehicle. We then crop the vehicle using the x-axis and y-axis coordinates of the square bounding box. We then scale the vehicular images and preserve the original aspect ratio. All the images in the dataset are flipped such that the vehicle is facing to the right, as the direction of the vehicle determined by a tracking algorithm applied to the original image sequence. The goal of this step is to remove any pose variability present in the data. The rest of the cropped image is padded with zeros. In Figure 3, we show an example for a vehicle image before and after cropping.

### 4.2. Wheel Detection

**Figure 4:**
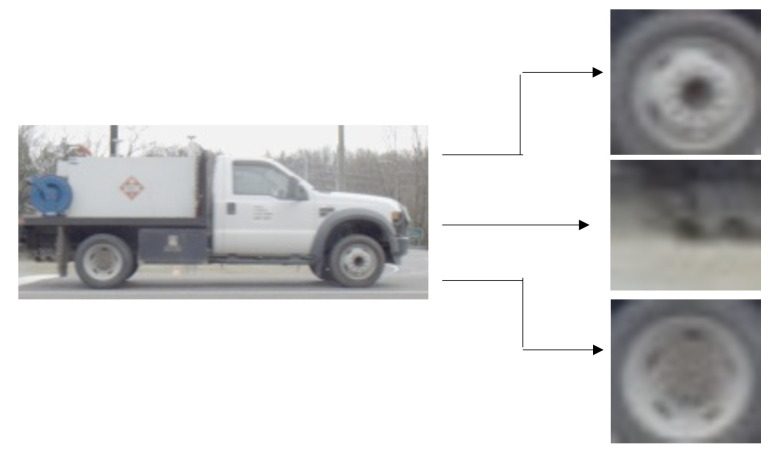
A Vehicle image and its detected wheels before wheel selection.

Similar to the vehicle detector, we trained a wheel detector to detect and locate vehicle wheels in video frames. We introduced an older version of the wheel detector in a previous paper [4]. The wheels were manually labeled for 4077 images using LabelImg [29]. The training set was constrained to represent diverse types of vehicles such as sedans, SUVs, trucks, vans, and big-rigs. We also included images that were taken under different lighting conditions. We subsequently retrained the SSD Mobilenet v2 network [27] to provide the bounding boxes information for vehicles’ wheels. Each detection is accompanied with a confidence score. In Figure 4, we show an example of detected wheels. From the figure, it can be concluded that some of the detections might not be accurate. In Section 4, we will elaborate on how we select the best candidate pair of wheels from the wheel detector output.

### 4.3. Wheel Selection

**Figure 5:**
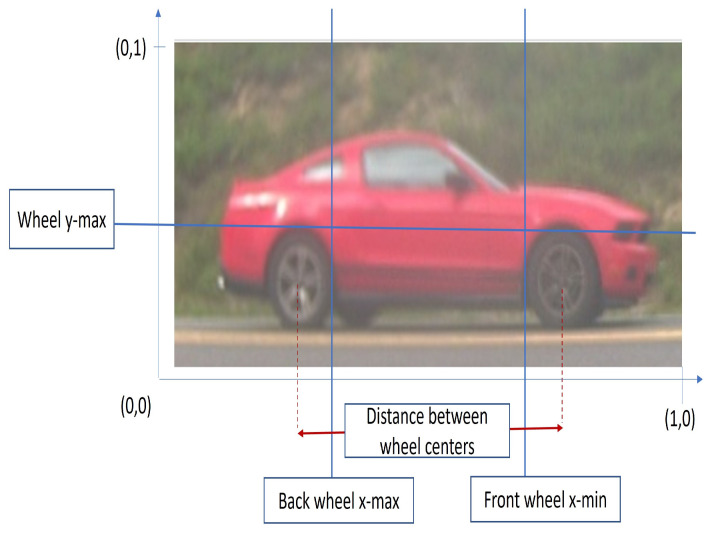
The measures utilized in our approach; y-axis coordinate, back wheel location, front wheel location, and the distance between wheel centers.

In the following section, we will elaborate on how we select the best candidate pair of wheels from the wheel detector output. Each wheel detection is represented by a bounding box. Each bounding box has 4 coordinates $\left[x_{left},y_{bottom},x_{right},y_{top}\right]$, where each coordinate is normalized between ∈[0,1]. We investigated the wheels location statistics using a portion of the dataset. We manually verified and labeled the wheel locations for 80,562 images representing 2128 different vehicles. In Figure 5, we show the four measures that are utilized in our approach to select the best pair of wheel candidates for each vehicle image.

**Figure 6:**
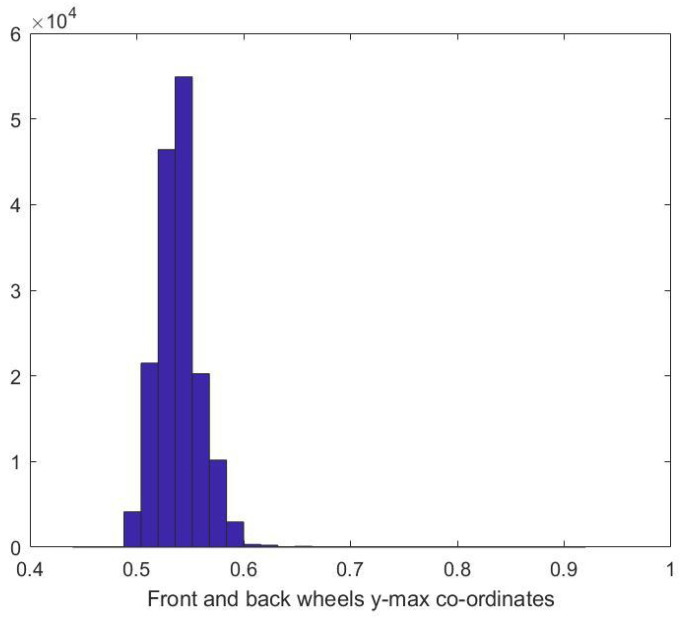
Wheels y-max co-ordinates histogram.

The first measure we considered is the vertical location of the wheels with respect to the vehicle bounding box. In Figure 6, the histogram of the $y_{top}$ coordinate. From the histogram, it can be inferred that the wheels lie in the lower half of the box.

**Figure 7:**
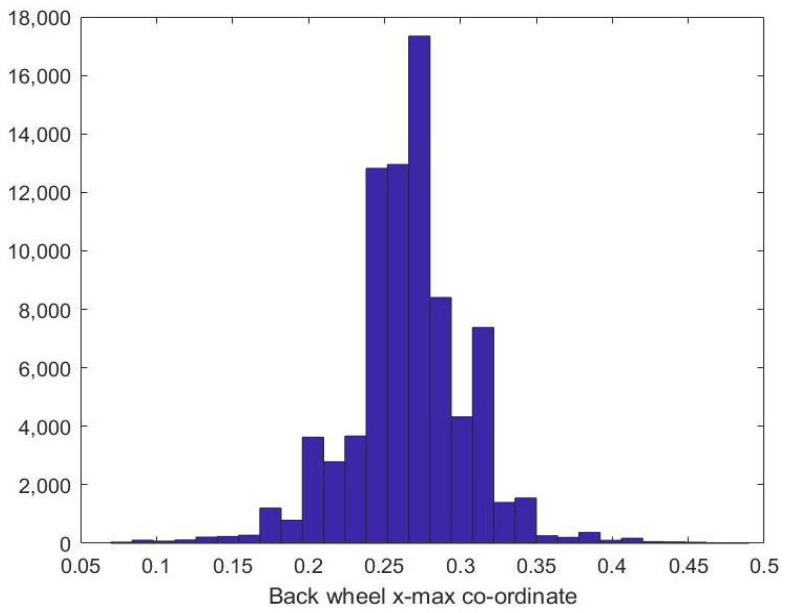
Back-wheel x-max co-ordinate histogram.

**Figure 8:**
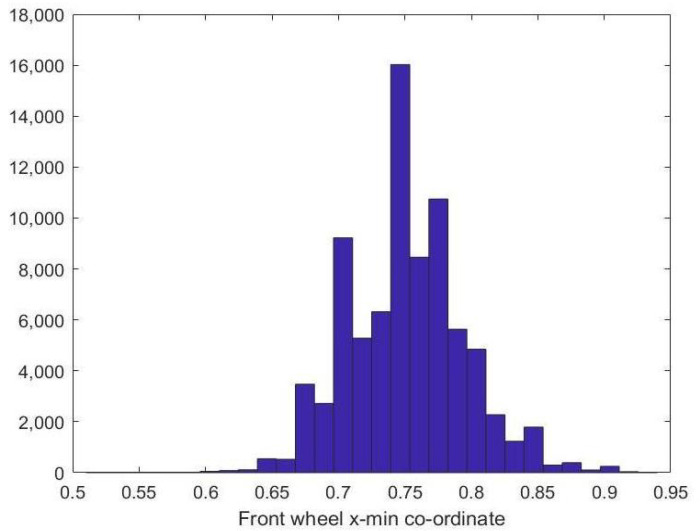
Front-wheel x-min co-ordinate histogram.

Next, we show the histograms of the wheels location on the x-axis. In Figure 7, the histogram of back wheel $x_{right}$ coordinate is demonstrated, while Figure 8 shows the histogram of front wheel $x_{left}$ coordinate. From the plots, it can be concluded that the back wheel typically lies in the back third of the image while the front wheel is located in the front third of the box, assuming the vehicle is moving from left to right.

**Figure 9:**
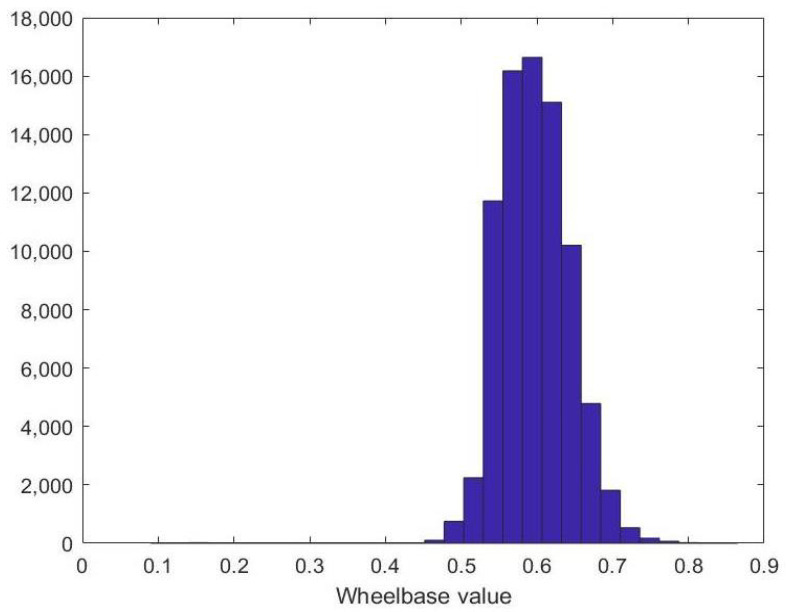
The distance between wheel-centers (wheelbase) histogram.

In addition to the wheels locations in the video frames, we also investigated the distance between wheel centers or wheelbase. The wheelbase is the horizontal distance between the centers of the front and rear wheels. The wheelbase is crucial for vehicle stability on the road and impacts the turning circle of a vehicle. Vehicles with a longer wheelbase are more stable at highway speeds but become more difficult to turn in tight turns. In Figure 9, the wheelbase histogram is demonstrated. From the the plot, it is clear that the wheelbase usually lies between 50% and 70% of the bounding box dimension. In our experiments, the bounding boxes’ coordinates are thresholded to filter out the wrong wheel detections and find the wheels that make sense physically. In particular, the thresholds applied on the $y_{top}$ coordinate and the wheelbase are optimized to achieve the highest wheel-detection accuracy. To that end, we utilized parameter optimization loops to iterate over different thresholds in reasonable ranges. This pre-processing step is referred to here as “Wheel Selection”.

In addition to thresholding the wheel coordinates, we also aligned the wheel detections. To that end, we used Random sample consensus (RANSAC) method to fit a line to those wheel centers having high confidence scores. RANSAC is an iterative approach that estimates a model from the data that has outliers. The RANSAC algorithm identifies the outliers in the data and predicts the model that fits noisy data. We subsequently filter out detections whose centers lie too far away from that line. This filtering step is here referred to as “Wheel alignment”.

### 4.4. Experimental Results

**Figure 10:**
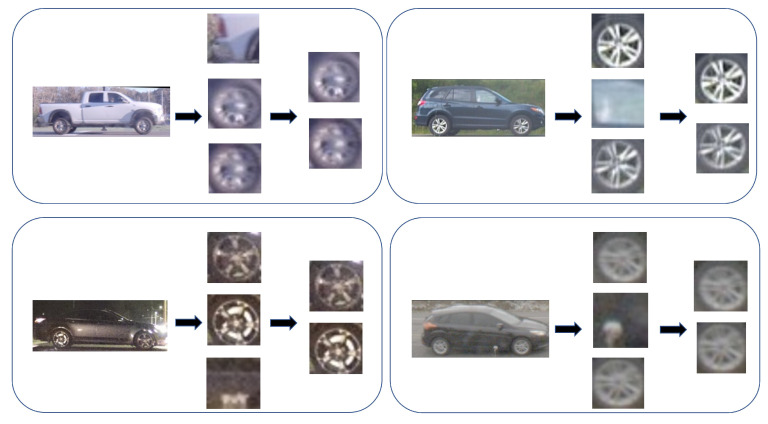
Vehicle images and their detected wheels before and after wheel selection.

In the following, the results of our wheel detection approach are shown. After cropping and resizing the vehicle images, we applied our wheel detector to provide the bounding boxes information for the wheels. We then filtered out the wrong wheel detections using the wheel selection and wheel alignment processes as explained in the previous section. In Figure 10, we show some examples of detected wheels after applying wheel selection and wheel alignment.

The validation set utilized has 80,562 images representing 2128 vehicles. Out of the 80,562 images, 76,547 images have two wheel detections or more. Each vehicle can have a different number of frames. In Table 1, we compare the detection performance of the wheel selection + wheel alignment approach versus only using wheel alignment.

To better explore the potential contribution of our proposed approach, we compare it with a baseline method. The baseline approach selects the two wheels with highest confidence in each image. In Figure 4, we show an example for three wheel detections with the highest confidence scores. The baseline method picks the first pair of images as the predicted wheel pair. Accuracy here is defined as the percent of images for which the Euclidean distance between the centers of the detected wheels bounding boxes $D_{P}$ and the ground truth detections $D_{GT}$ is less than 3% of the bounding box dimension, i.e., $(∥D_{GT}^{1}-D_{P}^{1}{∥}_{2}+∥D_{GT}^{2}-D_{P}^{2}{∥}_{2})<3\%$. The detected wheels bounding boxes might be slightly shifted to the left or to the right compared to the ground truth bounding boxes. In addition, the detected wheels’ bounding boxes can be bigger or smaller than the ground truth bounding boxes. As a result, we had to allow a tolerance for variations when the detection accuracy is measured, hence the 3% difference between the detected and ground truth box centers. Besides the number of images, we also report the number of vehicles having one or more frames with correct wheel detections. From the results, it can be concluded that utilizing our approach can precisely detect the wheel bounding boxes and retain a higher number of vehicles.

### 4.5. Vehicle Re-Identification

In this section, we will investigate the efficacy of the proposed wheel detector. The finer details from the wheels will be utilized to match a a pair of vehicles and produce a matching score. Based on the matching score, a decision is made either that is the same vehicle (True match) or if it is a different one (False match). The aim is to show that detected wheel patterns can be utilized to differentiate between vehicles. To that end, we trained a Siamese network [30] to match the front and rear wheels for each pair of vehicles. The wheel matching Siamese network, which we introduced in [4], consists of two identical branches. The two branches share the same parameters and network weights are updated similarly during training. The network computes the similarilty between the input wheel images by matching their signatures. Each branch consists of five convolutional layers. The input to each branch is a single wheel image that was resized to 100 × 100 × 3. Our proposed wheel detector estimates the bounding boxes for the front and rear wheels utilizing the mechanism described in Section 4.2 and Section 4.3, respectively. For validation, we compare the results of the vehicle re-ID using both the wheels detected by the baseline approach and our proposed wheel detector. Specifically, the detected wheels corresponding to each pair of vehicles in the validation set are cropped and matched using the Siamese model. The output feature vectors of the two branches is then fed into the last layer of the network, which produces the similarity score. The output score is a measure between 0 and 1. For each pair of vehicles, the front wheel of the first vehicle is compared to the front wheel of the other vehicle and similarly the rear wheels are also compared. A threshold is then applied to the average of the two wheel matching scores, i.e., if the average of the scores is more than 0.5, then the two vehicles are declared to be the same and vice versa. We compared the performance of vehicle re-ID using the baseline detector versus our proposed wheel detector and the results are shown in Table 2. From the results, it can be inferred that the proposed vehicle matching network that uses the wheels from our wheel detector is more reliable and accurate than the re-ID network that utilizes the wheels detected with the baseline method.

## 5. Conclusions and Future Work

In this paper, a wheel detection approach is proposed for reliably detecting wheel bounding boxes using deep neural networks and a vehicle-specific post-processing algorithm to eliminate false detections. This approach enables accurate re-centering of the vehicle image based on wheel coordinates for improved re-identification. Subsequently leveraging the wheel geometry can provide additional identifying information about the vehicle. A software for our wheel detection and post-processing approach is provided. We compared the performance of our framework with a conventional wheel detector that selected the pair of wheels with highest confidence. Experimental results demonstrate the efficacy of our proposed wheel detector under different illumination conditions and elevation angles. A limitation of this work is that it is applicable as long as the wheels are shown in the vehicle image, no matter what the shooting angle or the illumination conditions are. Proposed future work includes investigating the use of multi-view vehicular imagery for vehicle re-identification.

## Figures and Tables

**Table 1:** Wheel Detection Approach Performance. (Wheel Selection + Wheel Alignment vs. Wheel Alignment).

	Baseline	Wheel Alignment + Wheel Selection
Percentage of retained vehicles after wheel detection	98.78%	99.34%
Percentage of images with correct detections	77.03%	91.41%

**Table 2:** Vehicle re-ID performance using detected wheels.

	Baseline	Wheel Alignment + Wheel Selection
Vehicle re-ID performance	91.2%	92.02%

## Data Availability

Profile Images and Annotations for Vehicle Reidentification Algorithms (PRIMAVERA). Available online: http://doi.ccs.ornl.gov/ui/doi/367 (accessed on 1 January 2022), doi:10.13139/ORNLNCCS/1841347.
